# SOCIO-SPATIAL DETERMINANTS OF COMMUNITY-BASED LONG-TERM CARE AVAILABILITY IN RURAL COUNTIES

**DOI:** 10.1093/geroni/igae098.1819

**Published:** 2024-12-31

**Authors:** Claire Pendergrast

**Affiliations:** Syracuse University, Delmar, New York, United States

## Abstract

Community-based long-term care services are critical resources to support aging in place in communities across the country. These services may be especially important to rural older adults’ health and independence, as declining numbers of working-age adults in rural communities may reduce access to informal family supports. This study examines the spatial distribution of two forms of community-based long-term care in rural U.S. counties: aging and disability services organizations and home health agencies. Using data from the American Community Survey (ACS) and the National Neighborhood Data Archive (NaNDA) for 1708 rural counties, multi-level mixed-effects negative binomial models were estimated to examine the association between the availability of both forms of community-based long-term care services with county sociodemographic and sociocultural characteristics. Findings suggest significantly lower availability of aging and disability services per capita in rural counties with higher crime rates. Greater availability of these services was observed in more Republican counties, those with higher voter turnout, poverty rates, and shares of residents aged 65 and older, as well as shares of Black and Hispanic residents. Home health agency availability per capita was significantly greater in rural counties with higher crime rates, poverty rates, and shares of residents aged 65 and older. State-level factors explained 17.7% of the variation in rural counties’ aging and disability services availability and 4.5% of the variation in home health agency availability. Further investigation is needed to understand place-based factors driving inequalities in community-based long-term care access in rural areas.

